# Biochemical Characterization of Quinolinic Acid Phosphoribosyltransferase from *Mycobacterium tuberculosis* H37Rv and Inhibition of Its Activity by Pyrazinamide

**DOI:** 10.1371/journal.pone.0100062

**Published:** 2014-06-20

**Authors:** Hyun Kim, Keigo Shibayama, Emiko Rimbara, Shigetarou Mori

**Affiliations:** Department of Bacteriology II, National Institute of Infectious Diseases, Musashi-Murayama, Tokyo, Japan; Institute of Enzymology of the Hungarian Academy of Science, Hungary

## Abstract

Quinolinic acid phosphoribosyltransferase (QAPRTase, EC 2.4.2.19) is a key enzyme in the *de novo* pathway of nicotinamide adenine dinucleotide (NAD) biosynthesis and a target for the development of new anti-tuberculosis drugs. QAPRTase catalyzes the synthesis of nicotinic acid mononucleotide from quinolinic acid (QA) and 5-phosphoribosyl-1-pyrophosphate (PRPP) through a phosphoribosyl transfer reaction followed by decarboxylation. The crystal structure of QAPRTase from *Mycobacterium tuberculosis* H37Rv (MtQAPRTase) has been determined; however, a detailed functional analysis of MtQAPRTase has not been published. Here, we analyzed the enzymatic activities of MtQAPRTase and determined the effect on catalysis of the anti-tuberculosis drug pyrazinamide (PZA). The optimum temperature and pH for MtQAPRTase activity were 60°C and pH 9.2. MtQAPRTase required bivalent metal ions and its activity was highest in the presence of Mg^2+^. Kinetic analyses revealed that the *K_m_* values for QA and PRPP were 0.08 and 0.39 mM, respectively, and the *k_cat_* values for QA and PRPP were 0.12 and 0.14 [s^-1^], respectively. When the amino acid residues of MtQAPRTase, which may interact with QA, were substituted with alanine residues, catalytic activity was undetectable. Further, PZA, which is an anti-tuberculosis drug and a structural analog of QA, markedly inhibited the catalytic activity of MtQAPRTase. The structure of PZA may provide the basis for the design of new inhibitors of MtQAPRTase. These findings provide new insights into the catalytic properties of MtQAPRTase.

## Introduction

Tuberculosis (TB) is a chronic infectious disease, caused by the intracellular pathogen *Mycobacterium tuberculosis*, with an estimated 8.7 million cases and 1.4 million deaths each year according to a 2012 World Health Organization (WHO) Report [1]. The emergence of resistance to anti-TB drugs, in particular multidrug-resistant TB (MDR-TB), is a public health problem and poses a serious threat to global control of TB [2]–[4]. Therefore, there is an urgent need for new countermeasures against TB. To address this issue, the aim of the present study was to define the functions of poorly characterized enzymes that may provide targets for designing new drugs to eradicate *M. tuberculosis* infections.

Quinolinic acid phosphoribosyltransferase (QAPRTase; EC 2.4.2.19) is encoded by *nadC* and is a key enzyme in the *de novo* pathway of nicotinamide adenine dinucleotide (NAD) biosynthesis [5]–[7]. NAD is a coenzyme of pivotal importance in maintaining redox balance and energy metabolism and is continuously interconverted between oxidized (NAD) and reduced (NADH) forms [8]. In most bacteria, NAD biosynthesis is essential for cell survival and viability [9], which makes it an attractive target for the development of new antibacterial drugs, with steps shared by *de novo* and recycling pathways as a source of candidate enzymes for therapeutic intervention [5], [10]–[12].

**Figure 1:**
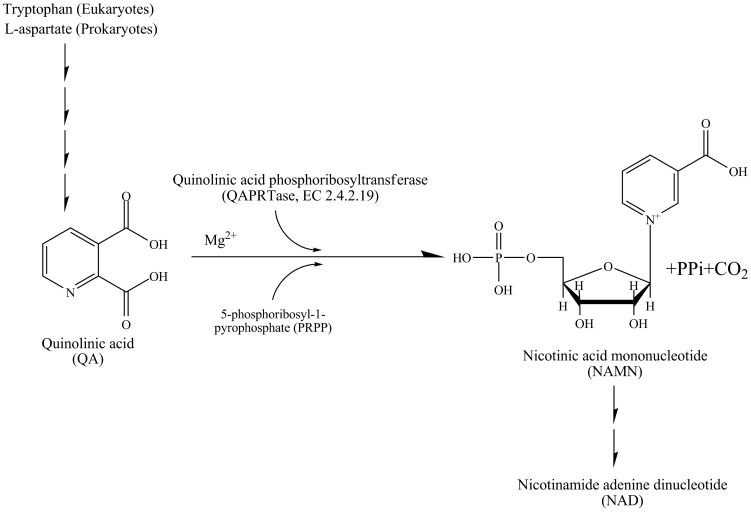
Schematic representation of the reaction catalyzed by QAPRTase.

QAPRTase catalyzes the Mg^2+^-dependent transfer of the phosphoribosyl moiety from 5-phosphoribosyl-1-pyrophosphate (PRPP) to the nitrogen atom of quinolinic acid (QA) to generate nicotinic acid mononucleotide (NAMN), pyrophosphate (PPi), and CO_2_ (Fig. 1) [5], [13]–[15]. QA is the first intermediate in the *de novo* pathway of NAD biosynthesis that is common to all organisms and is mainly produced by the degradation of tryptophan in most eukaryotes [5], [16], [17]. In contrast, in prokaryotes, including *M. tuberculosis*, it is produced from l-aspartate and dihydroxyacetone phosphate by the enzymes encoded by *nadA* (quinolinic acid synthetase) and *nadB* (l-aspartate oxidase) [18], [19]. In *M. tuberculosis*, *nadA, nadB*, and *nadC* are encoded in a single operon (*nadABC*) which is regulated by a repressor encoded by *nadR* [5], [20].

QAPRTase is a member of the phosphoribosyltransferase (PRTase) family of enzymes, which catalyzes the formation of nucleotides from nitrogenous bases and their common substrate PRPP [7], [13], [21]. PRTases are classified into four subclasses (types I, II, III, and IV) according to their structures [15], [22]–[26]. QAPRTase is a type II PRTase that participates in the *de novo* pathway of the pyridine coenzyme NAD [7], [15]. Recently, nicotinic acid phosphoribosyltransferase (NAPRTase) and nicotinamide phosphoribosyltransferase, which are involved in the salvage pathways of NAD biosynthesis, have been classified as type II PRTases [15], [23], [24], [27]. The activities of QAPRTase and NAPRTase were similar, although they are specific for their respective substrates [28], [29]. *M. tuberculosis* relies entirely on the *de novo* pathway of NAD for survival; therefore, it should be extremely vulnerable to drugs targeted against QAPRTase. The crystal structure of QAPRTase from *M. tuberculosis* (MtQAPRTase) is known [5]; however, the biochemical properties of MtQAPRTase remain to be determined. Therefore, in the present study, we examined and characterized the enzymatic activities of MtQAPRTase.

QA is a structural analog of the anti-tuberculosis prodrug pyrazinamide (PZA), and pyrazinoic acid (POA) is its active form. PZA is an important component of first line anti-TB drugs in the chemotherapy for TB and MDR-TB [30], [31]. Mycobacteria acquire resistance to PZA through mutations in the gene encoding pyrazinamidase (PZase), an enzyme that converts PZA to the active anti-bacterial form of POA [30], [32], [33]. Although mutations in PZase (encoded by *pncA*) responsible for the generation of most PZA-resistant *M. tuberculosis* strains have been identified [9], some PZA-resistant *M. tuberculosis* strains do not harbor *pncA* mutations [33]. The mechanism of action and main target of PZA are still not clearly understood; however, intensive investigations are in progress across laboratories worldwide [30]–[34]. Recently, Shi W. *et al*. [31] reported that the PZA inhibits trans-translation in *M. tuberculosis*, and suggest that POA binds to ribosomal protein S1 (RpsA) and subsequently inhibits trans-translation. Therefore, PZA may have interfered with bacterial growth and survival.

Because PZA and POA are structural analogs of QA, we reasoned that MtQAPRTase may use PZA or POA as a substrate, or these substrates may inhibit the enzymatic activity of MtQAPRTase.

In the present study, we examined and characterized the enzymatic activities of wild type (WT) plus mutant MtQAPRTases and the effect of PZA and POA on WT MtQAPRTase *in vitro*.

## Materials and Methods

### Materials

Pyrazinoic acid (POA), nicotinic acid mononucleotide (NAMN), 2,3-pyridinedicarboxylic acid (QA), and 5-phosphoribosyl-1-pyrophosphate (PRPP) were purchased from Sigma-Aldrich (Castle-Hill, Australia). Pyrazinamide (PZA) was from Tokyo Kasei Kogyo (Tokyo, Japan). Ammonium dihydrogen phosphate, t-butyl ammonium hydrogen sulfate, isopropyl β-d-1-thiogalactopyranoside (IPTG), ampicillin, and kanamycin were purchased from Wako Pure Chemicals Ltd (Tokyo, Japan). The TOPO TA cloning kit (pCR 4-TOPO) was purchased from Life Technologies (Carlsbad, CA) and used for cloning and nucleotide sequencing. The BCA protein assay kit, pH range 2.2–11.0 stock option pH buffer kit, and gel-filtration calibration kit were from Fisher Thermo Scientific (Pierce, Rockford, IL), Hampton Research (Aliso Viejo, CA) and GE Healthcare Bio-Sciences (Buckinghamshire, UK), respectively. Restriction endonucleases were purchased from New England BioLabs, Inc. (Ipswich, MA).

### Bacterial strains and plasmid

*Escherichia coli* strain DH5α (Life Technologies) was used as the host for molecular cloning. *E. coli* strain BL21 (DE3) was purchased from Merck KGaA (Darmstadt, Germany) and used for protein expression. The pET-30a plasmid (Merck KGaA) was used construct in an expression vector to produce WT and mutant versions of recombinant MtQAPRTase.

### Cloning and mutagenesis of *nadC* from *M. tuberculosis* H37Rv genomic DNA

Genomic DNA from *M. tuberculosis* H37Rv was isolated as previously described [35], [36]. The *nadC* (Rv1596, accession number; NP_216112.1) of *M. tuberculosis* H37Rv was amplified from genomic DNA [20] by using the polymerase chain reaction (PCR). The reaction mixture (20 µL) contained long and accurate (LA) PCR buffer II (Mg^2+^-free); 2.5 mM MgCl_2_; 200 µM each of dATP, dCTP, dGTP, and dTTP; 250 ng of genomic DNA from *M. tuberculosis* H37Rv; 1.25 units of LA Taq DNA polymerase (all from TaKaRa Bio, Kyoto, Japan); and 0.1 µM of each primer. The primers are shown in Table 1. PCR was conducted using a Takara PCR Thermal Cycler Dice Mini (TaKaRa Bio Inc., Shiga, Japan) as follows: pre-denaturation at 98°C for 2 min, 35 cycles of denaturation at 98°C for 10 sec, annealing at 55°C for 10 sec and extension at 72°C for 2 min, and final extension at 72°C for 2 min. K-001 and K-003 primers were used to amplify WT *nadC* (Table 1). Nucleotide sequences encoding a 6x-histidine residue cluster were added directly upstream of the *nadC* stop codon on C-terminal. The PCR product (885 bp) was ligated to the TA cloning plasmid and used to transform *E. coli* DH5α. The recombinant *nadC* plasmid DNA was recovered from the colonies and digested with *Nde*I and *Xho*I. The *nadC* product was ligated to pET-30a expression vector that was digested with the same restriction endonucleases. Mutant *nadC* genes were generated from WT *nadC* by using a QuikChange Site-Directed Mutagenesis Kit (Agilent Technologies, Inc., Santa Clara, CA) according to the manufacturer's instruction. The list of primers used to generate mutants is shown in Table 1. After mutagenesis, plasmids were recovered and purified using a Promega Minipreps DNA purification kit (Madison, WI, USA). WT and mutant plasmids were confirmed by sequencing with ABI Prism BigDye Terminator v3.1 Cycle Sequencing kit (Life Technologies). The sequencing reactions were performed according to the manufacturer's instructions. The sequencing products were analyzed using an ABI Prism 3130*xl* Genetic Analyzer (Life Technologies). The sequences generated by the software were compared with their respective *nadC* sequence using Bioedit software (http://www.bioedit.com/). Using the molecular modeling program MOE (Molecular Operating Environment, Chemical Computing Group, Montreal, Canada), we constructed a few models, which were elongated by six histidines at the C-terminus. These models were generated and structure energies were minimized using CHARMM27 force field (gradient below 0.5); the resulting structures were used as starting conformations for molecular dynamics simulations (MD) for 100 ps at 300 K. During the simulation, all amino acid residues were flexible.

**Table 1:** PCR primers.

Primer NO.	Sequence of oligonucleotide (Positions)	Comments
K-001	5′—CC*CATATG*GGGTTATCCGACTGGG—3′ (1–19)	WT *nadC*
K-003	5′—GG*AAGCTT*CTAATGATGATGATGATGATGCATATCCAAGCCGATGTC—3′ (838–858)	WT *nadC*
K-006	5′—CAACATGGTGGCCTCGGCGGTC—3′ (303–324)	Arg105Ala-Rv-*nadC*
K-007	5′—CCGCCGAGGCCACCATGTTGAAC—3′ (305–327)	Arg105Ala-Fw-*nadC*
K-008	5′—CAGCGTCTTAGCGGTATCGC—3′ (406–426)	Arg139Ala-Rv-*nadC*
K-009	5′—CGCGATACCGCTAAGACGCTGC—3′ (405–427)	Arg139Ala-Fw-*nadC*
K-010	5′—CCCAACCCCAGCGCATGGTTGAC—3′ (474–497)	Arg162Ala-Rv-*nadC*
K-011	5′—CGTCAACCATGCGCTGGGGTTGG—3′ (473–427)	Arg162Ala-Fw-*nadC*
K-012	5′—CGTGGTTGTCCGCGATTAGCGC—3′ (504–526)	Lys172Ala-Rv-*nadC*
K-013	5′—CGCGCTAATCGCGGACAACCACG—3′ (503–526)	Lys172Ala-Fw-*nadC*

Mutated codons are shown in bold type.

Restriction endonuclease cleavage sites and 6xHis are written in italics and underlined, respectively.

### Expression and purification of MtQAPRTase WT and mutants

WT and mutant forms of MtQAPRTase were purified as described previously [35], [37] with the following modifications. *E. coli* BL21 (DE3) was transformed with expression vectors carrying the *M. tuberculosis* WT or mutant *nadC*. Single colonies were picked and grown overnight at 37°C in 4 mL of Luria-Bertani (LB) medium containing 50 µg/mL kanamycin. Overnight cultures (2 mL) were used to inoculate 200 mL of LB medium containing kanamycin. Cells were then cultured at 37°C for 5 h, until the optical density (OD) at 600 nm reached 0.8 to 1.0. Expression of recombinant enzymes was induced with 1 mM IPTG, followed by incubation at 14°C for 18 h. The bacteria were harvested by centrifugation at 13,000×*g* at 4°C for 10 min, the pellets were stored at −80°C for 12 h, then suspended in 7 mL of binding buffer (20 mM sodium phosphate [pH 7.4], 0.5 M NaCl, 40 mM imidazole), and disrupted by sonication at 80% pulsar power, 40 sec on/1 min off 10-times on ice, using a UP50H sonicator (Hielscher Ultrasonic, Teltow, Germany). The extracts were centrifuged at 13,000×*g* at 4°C for 10 min, and the supernatants were harvested. The cell extracts were injected onto a HisTrap HP column (1.6×2.5 cm; GE Healthcare Bio-Sciences, Buckinghamshire, UK) pre-equilibrated with deionized water and binding buffer. The columns were washed with binding buffer until the absorbance reached a steady baseline, and the proteins were eluted using an elution buffer containing 20 mM sodium phosphate (pH 7.4), 0.5 M NaCl, and 0.5 M imidazole. The eluted proteins were concentrated using an Amicon Ultra-15 (Millipore, Billerica, MA) for 20 min at 4°C. WT and mutants MtQAPRTase were further purified using a Hi-Load 16/60 Superdex 200 prep grade column (GE Healthcare Bio-Sciences) in 10 mM sodium phosphate (pH 7.4), 50 mM NaCl buffer, and buffer exchange was performed to remove imidazole from the elutes with the same buffer. Based on the gel-filtration chromatography results, the eluted peak samples were assayed for QAPRTase activity and were analyzed using sodium dodecyl sulfate-polyacrylamide gel electrophoresis (SDS-PAGE). Protein concentrations were determined using a BCA Protein Assay kit.

### QAPRTase assay and characterization of MtQAPRTase activity

MtQAPRTase WT and mutant enzyme activities were determined by quantifying substrates using a high-performance liquid chromatography system (HPLC, Shimadzu, Kyoto, Japan) according to previously described methods with modifications [16], [35], [38], [39]. The HPLC system comprised a Prominence UV/VIS detector and a 4.6×250 mm COSMOSIL PACKED 5C18 AR-II column (Nacalai Tesque, Inc., Kyoto, Japan). Reaction mixtures contained 50 mM KH_2_PO_4_ (pH 7.2), 6 mM MgCl_2_, 1.5 mM QA, 1 mM PRPP, and 1.62 µM of purified WT or mutant MtQAPRTase in a total volume of 100 µL, and were then incubated at 37°C for 30 min; the reaction was stopped by heating at 98°C for 3 min, and then the reaction mixtures were centrifuged at 13,000×*g* for 5 min. The supernatants (100 µL) were subjected to HPLC analysis using a column equilibrated with deionized water. The adsorbed products such as QA, PRPP, and NAMN, were eluted with a gradient of water and HPLC buffer (0.02 M t-butyl ammonium hydrogen sulfate and 0.2 M dihydrogen phosphate [pH 5.3]) at a flow rate of 0.6 mL/min, and products were detected by their absorbance at 270 nm. One unit (U) of enzyme activity was defined as the 1.0 µmol of NAMN in 1 min at 37°C. The effects of pH on enzyme activity were determined using citric acid anhydrous (pH 2.2 and 3.2), sodium acetate trihydrate (pH 4.2), TRIS-sodium citrate dehydrate (pH 5.2), cacodylic acid sodium salt trihydrate (pH 6.2), sodium 4-(2-hydroxyethyl)-1-piperazineethanesulfonic acid (pH 7.2), tri-hydrochloride (pH 8.2), and 3-(cyclohexylamino)-2-hydroxy-1-propane sulfonic acid (pH 9.2, 10.2, and 11.2). To determine the temperature optimum, QAPRTase activities were measured over the range of 20−70°C in 50 mM KH_2_PO_4_ (pH 7.2) for 30 min. The effects of divalent cations on QAPRTase activity were determined using reaction mixtures containing 6 mM each of Mg^2+^ (MgCl_2_), Mn^2+^ (MnCl_2_), Co^2+^ (CoCl_2_), Fe^2+^ (FeCl_2_), Ca^2+^ (CaCl_2_), Zn^2+^ (ZnCl_2_), and Cu^2+^ (CuSO_4_). Enzyme analyses were performed in triplicate to determine reproducibility.

### Kinetic parameters of MtQAPRTase

The kinetic parameters of recombinant MtQAPRTase activities were performed by a monitoring spectrophotometric assay using Ultrospec 3000 UV/Visible Spectrophotometer (GE Healthcare Biosciences, Buckinghamshire, UK). Typical assay mixtures contained 50 mM KH_2_PO_4_ (pH 7.2), 6 mM MgCl_2_, various concentration of QA (0.02–0.4 mM), PRPP (0.02–1.5 mM), and 1.62 µM of purified WT MtQAPRTase in a total volume of 100 µL. A spectrophotometric assay of QAPRTase activity over 20 min at 37°C measured the increase in absorbance at 266 nm resulting from the conversion of QA to NAMN (ΔA266  = 920 M cm^−1^) [16], [39]. Ultrospec 3000 UV/Visible Spectrophotometer plotted the increase in absorbance at 266 nm (dA) against time (min) and calculated the initial slope (dA/min) automatically. The initial velocity was calculated from the slope (dA/min). The reaction mixtures for determination of *K_m_* values from initial velocity data were prepared using various concentrations of PRPP and a fixed concentration of QA (0.3 mM); conversely, reaction mixtures were also prepared using various concentrations of QA and a fixed concentration of PRPP (1.0 mM). The reaction was initiated by the addition of WT MtQAPRTase, and absorbance was immediately estimated. To determine *K_m_* and *k_cat_* values when using QA and PRPP as substrates, their concentrations were varied. Kinetic parameters for MtQAPRTase activity were calculated using GraphPad Prism 5 software (GraphPad Software, La Jolla, CA). Values of *K_m_* and *k_cat_* are represented as mean ± standard error of three independent determinations.

### Inhibitory effects of PZA/POA and determination of IC_50_ values

The inhibitory effects of PZA and POA on MtQAPRTase activity were determined by quantifying substrates using a HPLC analysis. Reaction mixtures (100 µL) containing 6 mM MgCl_2_, 1.5 mM QA, 1 mM PRPP, 1 mM of PZA or POA, and 1.62 µM of purified WT MtQAPRTase were incubated at pH 6.2 or 7.2 in 50 mM KH_2_PO_4_ buffer at 37°C for 30 min. Additionally, the drug concentrations required to inhibit the QAPRTase activity by 50% (IC_50_ values) were determined from the concentration of NAMN generated at various concentrations of PZA (0.07–0.7 mM at pH 7.2 and 0.2–2.0 mM at pH 6.2) or POA (0.5–5.0 mM at pH 7.2 and 2.0–40.0 mM at pH 6.2). IC_50_ values were represented as mean ± standard error of three independent determinations.

### Determination of the molecular mass of MtQAPRTase

The molecular mass of purified MtQAPRTase was estimated using a Superdex 200 10/300GL (10×300 mm) (GE Healthcare Bio-Sciences) gel-filtration chromatography column with low- and high-molecular-weight (LMW, HMW) calibration kits (GE Healthcare Bio-Sciences) as recommended by the manufacturer. The protein standards and molecular masses were as follows: ferritin (440.0 kDa), aldolase (158.0 kDa), conalbumin (75.0 kDa), ovalbumin (44.0 kDa), carbonic anhydrase (29.0 kDa), ribonuclease A (13.7 kDa), and aprotinin (6.5 kDa).

### Molecular docking study

The molecular docking and visualization studies were performed using MF mypresto v1.2 (Fiatlux Corporation, Tokyo, Japan) molecular modeling software. Coordinates of MtQAPRTase for structure-based molecular modeling were retrieved from the Protein Data Bank (PDB), USA (http://www.rcsb.org/pdb/), under the accession code [PDB ID: 1QPQ] [5]. The molecular docking models used dimeric forms of A and B subunits from the three-dimer form of MtQAPRTase and the coordinates of ligands and waters were manually removed. On the basis of the information regarding the QA-binding site, the location and size of the receptor pocket were set (Center: X: -15.796 Y: 42.865 Z: 17.445, and radius: 4 angstrom). Optional parameters in MF mypresto v1.2 were used to create a topology file, which included addition of hydrogen atoms, calculation of a grid potential, and docking simulation. Flexible docking method was used and the scores are expressed as a sum of five potentials: accessible surface area, coulomb potential, hydrogen bonds, hydrogen bond considering anisotropy, and van der Waals interactions. The protein-ligand binding free energies were estimated by MF mypresto v1.2. The results of molecular docking were visualized, and the distance between residues of amino acid and PZA was calculated using PyMOL v1.3 (http://www.pymol.org/) and WinCoot-0.7.2 (http://www.ysbl.york.ac.uk/).

## Results

### Expression and purification of MtQAPRTase

**Figure 2:**
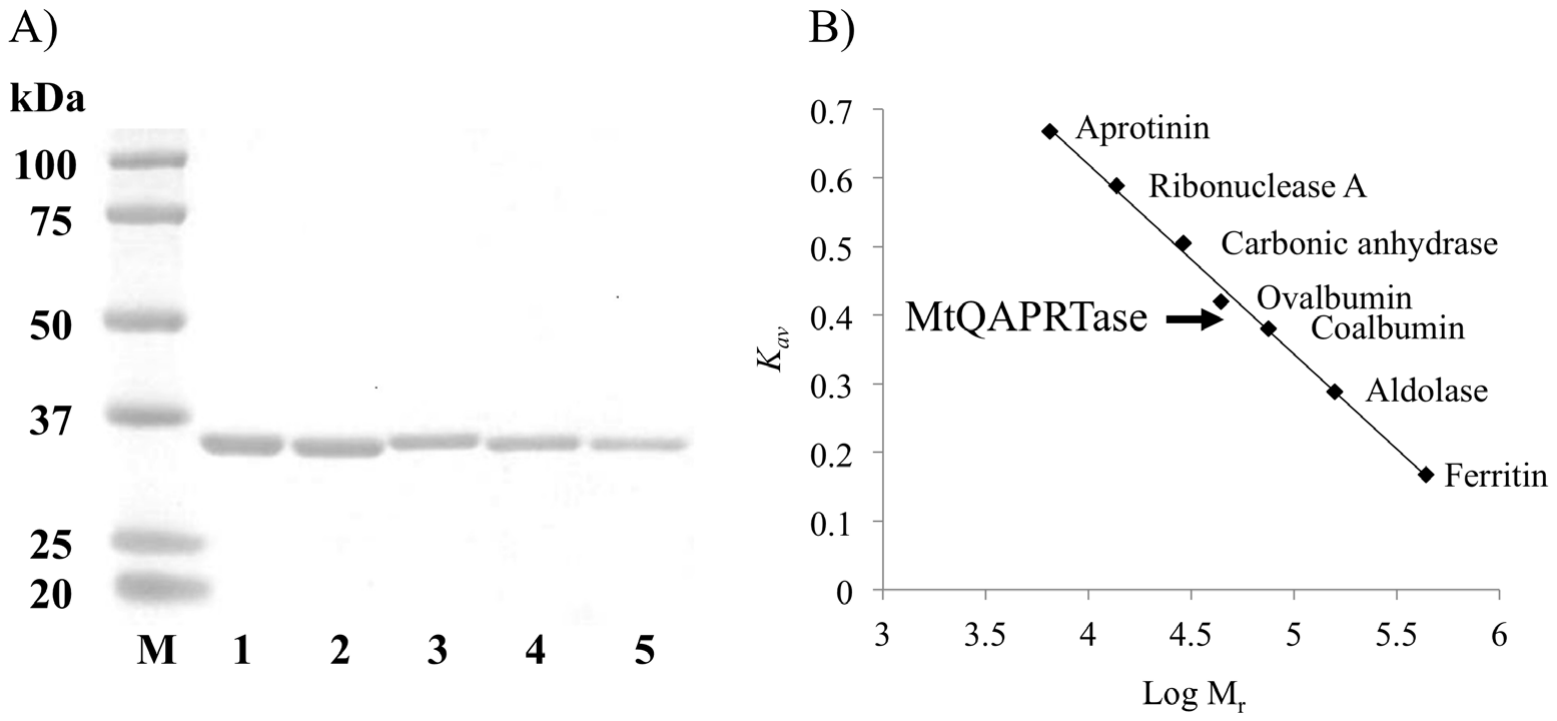
SDS-PAGE analysis and determination of the molecular mass of recombinant MtQAPRTases. A) The WT and mutant enzymes are shown above the designated lane. Approximately 5 µg of each protein was loaded on a 5–20% SDS-polyacrylamide gel. Lane M, protein size markers; lane 1, WT MtQAPRTase; lane 2, Arg105′-Ala; lane 3, Arg139Ala; lane 4, Arg162Ala; and lane 5, Lys172Ala. B) Estimation of the molecular mass of recombinant MtQAPRTase using Superdex 200 gel-filtration chromatography. The elution position of MtQAPRTase is indicated by an arrow. Protein standards were as follows: aprotinin (6.5 kDa); ribonuclease A (13.7 kDa); carbonic anhydrase (29.0 kDa); ovalbumin (44.0 kDa); conalbumin (75.0 kDa); aldolase (158.0 kDa), and ferritin (440.0 kDa). *K_av_* values were calculated using the following equation: *K_av_*  =  (*V_e_−V_o_*)/(*V_c_−V_o_*) is the column void volume, *V_e_* is the elution volume, and *V_c_* is the geometric column volume. The *V_o_* value used was the *V_e_* of Blue Dextran 2000. *M_r_* indicates the molecular weight.

Full-length *nadC* from *M. tuberculosis* H37Rv was inserted into the expression vector pET-30a downstream of the T7 promoter to express a His-tagged recombinant MtQAPRTase. We confirmed the association between the location of His-tag and active site of MtQAPRTase by using MOE. This analysis shows that the His-tag could be located outside the active site of MtQAPRTase (Fig. S1), suggesting that the His-tag does not interfere with the activity of MtQAPRTase. Furthermore, it was confirmed that His-tag could not affect the multimerization of MtQAPRTase (Fig. S1). DNA sequence analysis of the recombinant plasmid confirmed the identity and integrity of *nadC* and verified that no mutations were introduced during PCR amplification. Recombinant MtQAPRTase was purified to homogeneity using a two-step column chromatographic procedure described in Materials and Methods. The molecular mass of recombinant MtQAPRTase determined using SDS-PAGE was 31 kDa (Fig. 2A, lane 2), consistent with that calculated from the amino acid sequence containing six histidine residues (30773.9 Da). The specific activity of purified recombinant MtQAPRTase was 1.2 U/mg with a yield of 19% and purification of 6.4-fold (Table 2). The molecular mass of MtQAPRTase as estimated using gel-filtration chromatography column was approximately 58 kDa (Fig. 2B), indicating that the enzyme exists as a dimer in solution.

**Table 2:** Purification of recombinant QAPRTase from *M. tuberculosis* H37Rv.

Purification Step	Total Protein (mg)	Total activity (Unit)	Yield (%)	Specific activity (Unit/mg)	Purification (fold)
Cell extract	481.8	91.5	100	0.2	1.0
His-Trap	34.3	62.7	68	1.8	9.6
Sephacryl S-200	14.0	17.1	19	1.2	6.4

### Enzymatic activities of MtQAPRTase

**Figure 3:**
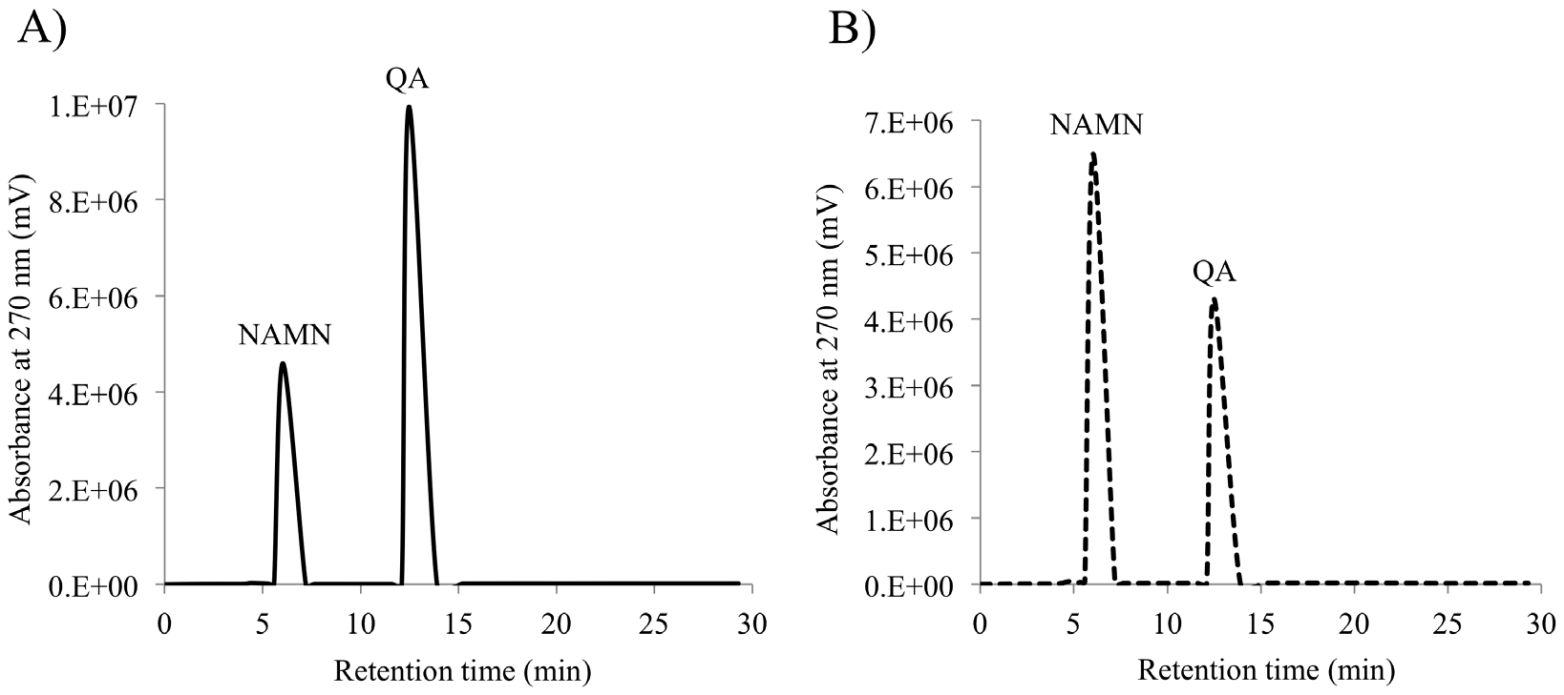
HPLC analysis of substrates and products. The enzymatic activities of MtQAPRTase were determined using QAPRTase assays with PRPP as the substrate in the absence (A) and presence (B) of purified recombinant MtQAPRTase. NAMN and QA eluted at 6.0 and 12.5 min.

The enzymatic activities of MtQAPRTase were determined using HPLC (Fig. 3). QA and NAMN were observed at 6.0 and 12.5 min, respectively (Fig. 3A), while a peak of PRPP was not detected using our separation conditions. When MtQAPRTase was added to a reaction mixture, the area of the QA and NAMN peaks decreased and increased, respectively (Fig. 3B). We then confirmed that recombinant MtQAPRTase converts QA and PRPP to NAMN, PP_i_, and CO_2_ in the presence of Mg^2+^.

**Figure 4:**
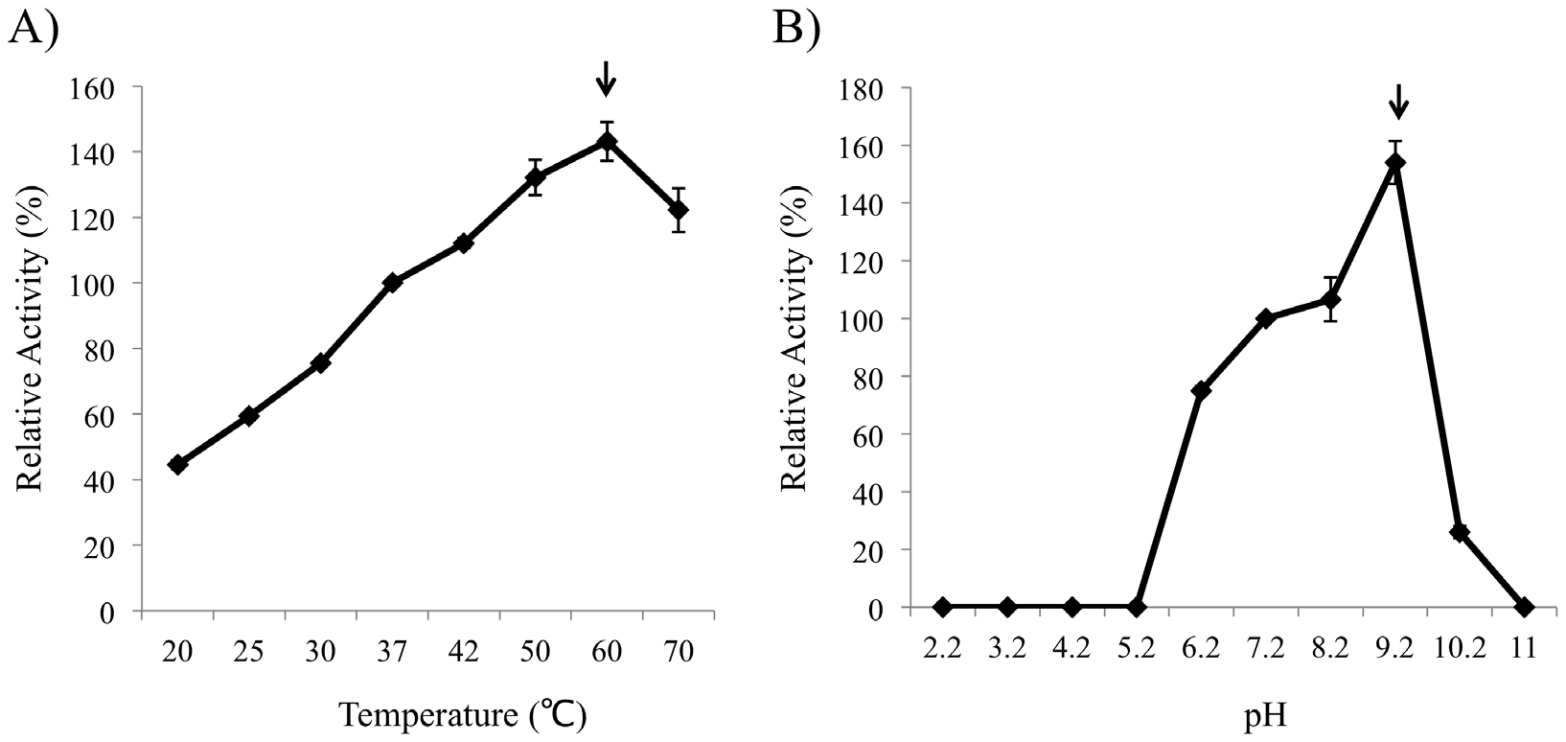
Temperature and pH optima. MtQAPRTase was incubated at the various temperatures (A) and at pH 2.2–11.0 (B) for 30 min. The optimum level of MtQAPRTase activity is denoted with arrows.

The optimum temperature of MtQAPRTase was 60°C, and its activity was decreased at 70°C (Fig. 4A). Its pH optimum was 9.2, and its activity could not be detected at pH values below 5.2 and above 11.0 (Fig. 4B).

QAPRTase isolated from *M. tuberculosis*, *E. coli*, and *Salmonella typhimurium* requires Mg^2+^ for activity [12], [15], [18]. Similarly, MtQAPRTase activity requires bivalent metal ions such as Mg^2+^. Activity was highest in the presence of Mg^2+^ (Table 3). The relative activities of MtQAPRTase in the presence of 6 mM metal ions are shown in Table 3.

**Table 3:** Effects of bivalent metal ions on QAPRTase activity.

Bivalent Metal Ions (6 mM)	Specific activity (Unit/mg)	Relative Activity (%)
Mg^2+^ (MgCl_2_)	1.32	100
Mn^2+^ (MnCl_2_)	0.35	27
Co^2+^ (CoCl_2_)	0.15	12
Fe^2+^ (FeCl_2_)	0.06	6
Ca^2+^ (CaCl_2_)	0	0
Zn^2+^ (ZnCl_2_)	0	0
Cu^2+^ (CuSO_4_)	0	0
None^a^	0	0

^a^absence of metal ions.

### Kinetic parameters of WT MtQAPRTase

**Figure 5:**
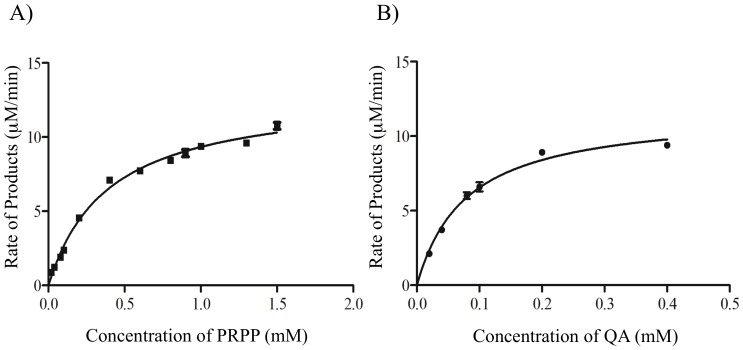
Kinetic studies of MtQAPRTase. The Michaelis–Menten plots for enzyme activity were generated in the presence of different concentrations of PRPP (A) and QA (B) as shown in the figure. Reaction mixtures (50 mM KH_2_PO_4_ [pH 7.2], 6 mM MgCl_2_, various concentrations of QA or PRPP, and 1.62 µM of MtQAPRTase) were incubated at 37°C over 20 min. Kinetic studies were performed using reaction mixtures that contained various concentrations of PRPP and a fixed concentration of QA (0.3 mM) as the substrate; conversely, kinetic studies were also performed using reaction mixtures that contained various concentrations of QA and a fixed concentration of the PRPP (1.0 mM) as the substrate. Standard error for three independent experiments is indicated by the bars.

The Michaelis–Menten plot was used to estimate *K_m_* and *k_cat_* according to the activity at different concentrations of PRPP (between 0.02 and 1.5 mM) and QA (between 0.02 and 0.4 mM) as substrates (Fig. 5). The *K_m_* and *k_cat_* values for PRPP were 0.39±0.03 mM and 0.14±0.30 [s^−1^], respectively (Fig. 5A). In contrast, the *K_m_* and *k_cat_* values for QA were 0.08±0.014 mM and 0.12±0.4 [s^−1^], respectively (Fig. 5B).

### Site-directed mutagenesis and activities of mutant MtQAPRTases

**Figure 6:**
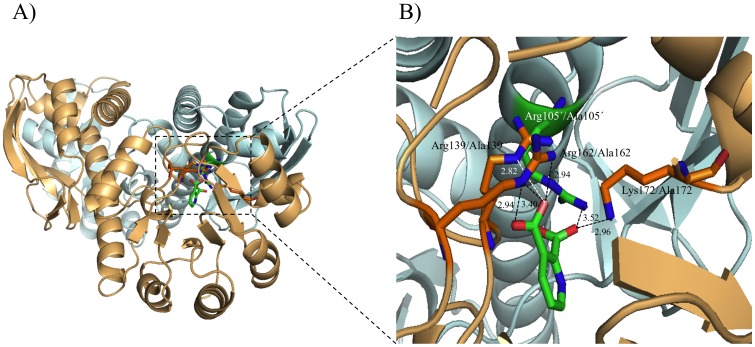
The QA binding mode in MtQAPRTase. The binding mode of QA is shown within the catalytic site of MtQAPRTase and the mutated amino acids (Arg105′Ala, Arg139Ala, Arg162Ala, Lys172Ala, and His175Ala) used in this study are shown. The A subunit of MtQAPRTase is depicted in light orange while the B subunit is depicted in pale cyan. The overall structures and the catalytic site on MtQAPRTase are shown on the left and right, respectively. The dotted line indicates hydrogen bonding, and the distance between amino acid residues and QA is indicated as well.

X-ray crystallographic studies of QAPRTase indicate that Arg105′, Arg139, Arg162, Lys172, and His175 are located in the QA-binding site of MtQAPRTase (Fig. 6) [5], [13]–[15], [40]. Arg105′ is present in another subunit. To determine whether these residues play an important role in the enzymatic activity of MtQAPRTase, we constructed, expressed, and purified the following MtQAPRTase-single mutants: Arg105′Ala, Arg139Ala, Arg162Ala, Lys172Ala, and His175Ala (Fig. 2A and 6). The catalytic activities of the MtQAPRTase mutants could not be detected when purified preparations were evaluated at variable concentrations up to 38.9 µM, which was more than 24-fold higher than normal condition (1.62 µM). Therefore, these results indicate that these residues play important roles in QA binding and catalysis of QAPRTase activity.

### Inhibitory effects of MtQAPRTase activity by PZA/POA and IC_50_ values

**Figure 7:**
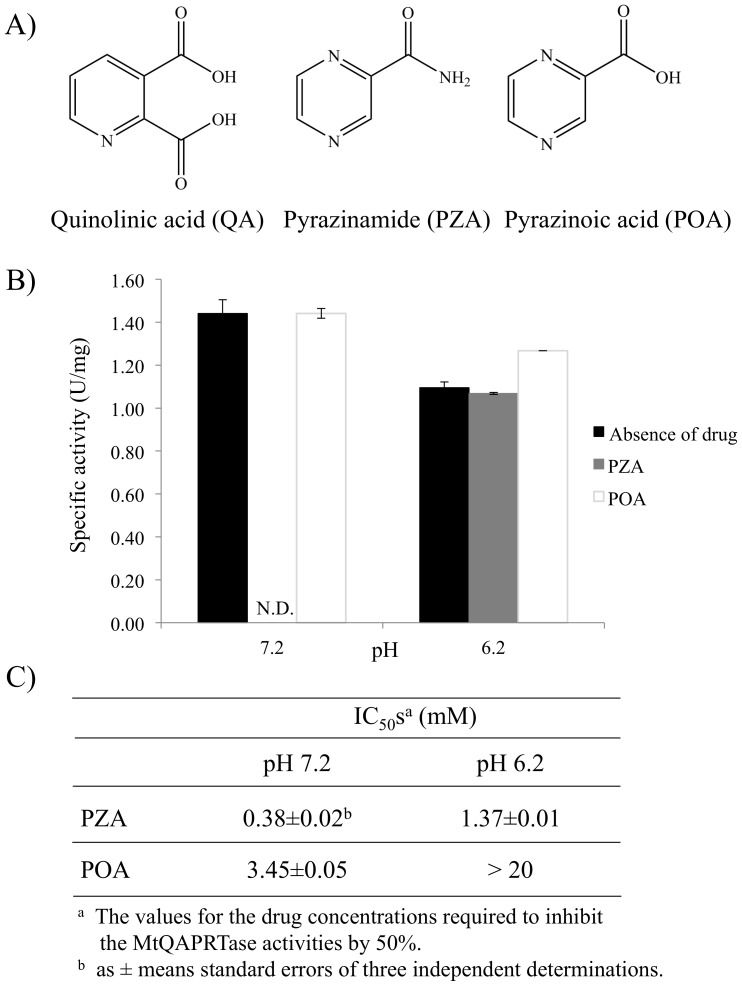
Inhibitory effect of PZA or POA on MtQAPRTase activity and the calculated IC_50_ values. (A) Chemical structures of QA, PZA, and POA are shown. Either 1 mM of PZA or POA was incubated with MtQAPRTase at pH 7.2 (B, left) and pH 6.2 (B, right) at 37°C for 30 min. After incubation, reactions were stopped and the products analyzed using HPLC. The IC_50_ values are indicated in (C). N.D. mean not detected. Data from three separate experiments are represented as mean ± standard error.

Because PZA and POA are structural analogs of QA (Fig. 7A), we predicted that these molecules may be substrates or inhibitors of MtQAPRTase. When PZA or POA were added to reaction mixtures instead of QA or PRPP, no new peaks were detected (data not shown), indicating that they are not substrates. To determine the inhibitory effects of PZA and POA on MtQAPRTase activity, PZA or POA was incubated with MtQAPRTase under different pH conditions (pH 7.2 and 6.2). Initially, we investigated the concentrations of PZA and POA under neutral (pH 7.2) and weak acidic (pH 6.2) conditions. PZA and POA were tested for their abilities to inhibit MtQAPRTase between the concentrations of 0.1 and 3.0 mM (data not shown). Figure 7B shows the results of a representative inhibitory QAPRTase assay containing 1 mM of PZA or POA at pH 7.2 (Fig. 7B left) and pH 6.2 (Fig. 7B right), respectively. Interestingly, the inhibitory effect of PZA at pH 7.2 condition was dramatically higher than at pH 6.2 condition and that of POA at any pH value. IC_50_ values of PZA and POA are summarized in Fig. 7C. The IC_50_ values of PZA were 0.38±0.02 and 1.37±0.01 mM at pH 7.2 and 6.2, and those of POA were 3.45±0.05 and >20 mM at pH 7.2 and 6.2, respectively. Using the IC_50_ values, the *K*_i_ values were estimated by a web-based tool [41], (http://botdb.abcc.ncifcrf.gov/toxin/kiConverter.jsp). The *K*_i_ values of PZA were 19.2 µM and 69.4 µM at pH 7.2 and 6.2, respectively, and that of POA was 174.7 µM at pH 7.2.

### Molecular docking study

**Figure 8:**
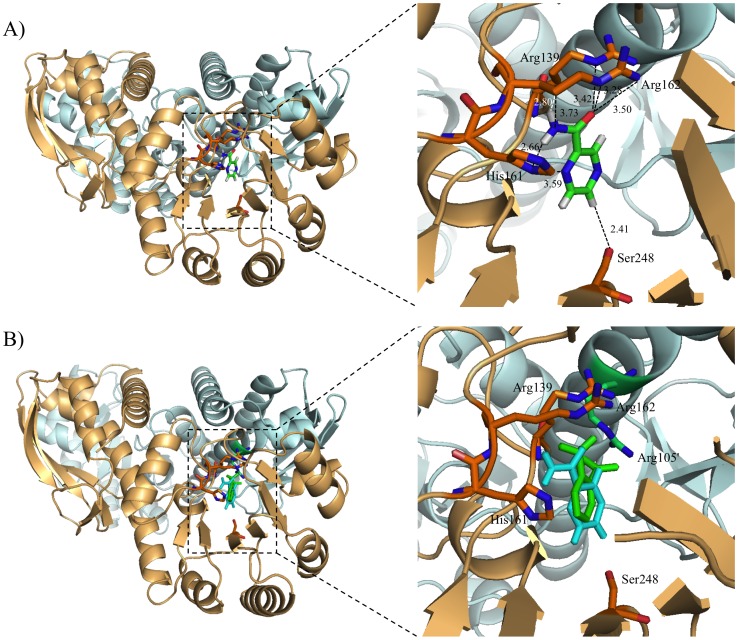
Molecular docking studies. The binding mode of PZA or QA was shown within the catalytic site of MtQAPRTase. The A subunit of MtQAPRTase is depicted in light orange while the B subunit is depicted in pale cyan. The overall structures and the catalytic site on MtQAPRTase are shown on the left and right, respectively. The models of complex structures are indicated as follows: (A) WT MtQAPRTase–PZA and (B) WT MtQAPRTase–merging with PZA [cyan] and QA [green]. The dotted line indicates hydrogen bonding (A and B), and the distance between amino acid residues and PZA/QA is indicated as well.

According to the values of the protein-ligand binding free energy and the score, the optimum docking model was selected. When the PZA was used as the ligand in molecular docking, the values of the protein-ligand binding free energy and the score were −5.22 and −1.80, respectively. On the other hand, when the QA was used as the ligand, the values of the protein-ligand binding free energy and the score were −5.87 and −2.07, respectively. There was no significant difference between the values of the protein-ligand binding free energy and the score of PZA and QA. Molecular docking results are shown in Fig. 8. Although the structure of QAPRTase has been reported as a three-dimer form [5], [16], we used dimeric forms of A and B subunits for the molecular docking study. Our docking results showed that PZA binds to Arg139, His161, Arg162, and Ser248 through a hydrogen-bonding network (Fig. 8A and Table 4). Arg139 and Arg162 are involved in QA binding too (Fig. 6) [5]. Therefore, we suggest that the PZA-binding site of MtQAPRTase overlaps the QA-binding site (Fig. 8B). Arg139, His161, Arg162, and Ser248 were changed to Ala after PZA was docked to MtQAPRTase by using the WinCoot-0.7.2 program. We found that Ala139, Ala161, Ala162, and Ala248 did not interact with PZA (data not shown); thus, we believe that these amino acid residues play an important role in the interaction between MtQAPRTase and PZA (Fig. 8)

**Table 4:** Selected contact between MtQAPRTase and PZA.

PZA atom	Protein atom	Distance (Å)
O	Arg139 NE	3.42
N1	His161 NE2	3.75
N1	His161 ND1	3.56
H2	His161 ND1	2.66
N3	His161 ND1	3.59
O	Arg162 NH2	3.50
O	Arg162 NE	3.28
H1	Arg162 CG	2.80
N1	Arg162 CG	3.73
H5	Ser248 OG	2.41

## Discussion

We describe here the molecular cloning, expression, and purification of MtQAPRTase to determine its biochemical properties. MtQAPRTase forms a dimer in solution, which is consistent with its crystal structure [5]. The optimum temperature and pH of MtQAPRTase were 60°C and pH 9.2 and are similar to those of the QAPRTase from *Alcaligenes eutrophus nov*. subsp. *Quinolinicus* [42]. Because the enzymatic activity of MtQAPRTase was detected at pH 7.2 and 37°C (Fig. 4), we suggest that MtQAPRTase functions as a QAPRTase in *M. tuberculosis*.

Liu H. *et al*. [16] reported the kinetic characterization of QAPRTase (hQAPRTase) from *Homo sapiens*. We compared the kinetic studies of MtQAPRTase with those of hQAPRTase and recalculated the values of *k_cat_* from *V_max_* values. The *k_cat_* value for QA determined using the MtQAPRTase assay was 0.12 [s^−1^] and that reported for hQAPRTase was 0.04 [s^−1^]. The *K_m_* values for QA when evaluating MtQAPRTase and hQAPRTase were 80 and 22 µM, respectively. Therefore, we suggest that the enzymatic activity of MtQAPRTase is similar to that of hQAPRTase.

The inhibitory effects of PZA or POA on MtQAPRTase activity were determined at neutral (7.2) and weak acidic (6.2) pH, because it was reported that in *M. tuberculosis*, at an acidic external pH. In *M. tuberculosis*, at an acidic external pH, the rate of passive transmembrane equilibrium of POA apparently overwhelms that of active efflux, resulting in a huge accumulation of POA in the cells [31], [43]. Although the internal pH in *M. tuberculosis* exposed to an acidic external pH is not known, we speculate that the internal pH is weakly acidic. Therefore, we evaluated the inhibitory effects at pH 6.2. Our results show that the inhibitory effect of PZA at neutral pH condition was dramatically higher than the inhibitory effect of POA at any pH value (Fig. 7B). Therefore, we suggest that PZA may inhibit the MtQAPRTase activity at neutral pH.

Structural studies indicate that Arg105′, Arg139, Arg162, Lys172, and His175 residues interact with QA, suggesting that the C3 carboxylate group of QA forms hydrogen bonds with the side-chain atoms of Arg162 and Arg139, whereas the C2 carboxylate group is within hydrogen-bonding distance of the main chain of Arg139 and the side chains of Arg105′ and Lys172 (Fig. 6) [5]. Further, the side chain of His161 is within van der Waals distance of the substrate [5]. We found that the four mutated amino acids play an important role in enzymatic activity of MtQAPRTase. QA binds tightly to QA-binding site (Arg105′, Arg139, Arg162, and Lys172) through hydrogen bonds (H-N-H―O-H and H-N―O-H) and other bonds (Fig. 6) [5], but not to alanine mutants (Ala105′, Ala139, Ala162, and Ala172) because of the absence of hydrogen-bonding (data not shown). The subsequent loss of hydrogen bonding could induce a substantial conformational change, which disrupts enzymatic activity of MtQAPRTase.

We further demonstrated that PZA strongly inhibited MtQAPRTase activity (Fig. 7). Based on the crystal structure and data on the interactions between QA and QAPRTase [5], [13], we hypothesize that the PZA-binding site of QAPRTase overlaps the QA-binding site, because the structures of PZA and QA are very similar (Fig. 7A). Therefore, we investigated the binding mode of a PZA instead of a QA within the active site of MtQAPRTase using molecular docking analysis (Fig. 8). Our molecular docking results show that the PZA binds to amino acid residues (Arg139, His161, Arg162, and Ser248) through a hydrogen-bonding network (Fig. 8A and Table 4), and QA binds to amino acid residues (Arg105′, Arg139, Arg162, and Lys172) on MtQAPRTase (Fig. 8B) [5]. We suggest further that Arg139 and Arg162 are essential amino acid residues for the formation of QA- and PZA-binding sites, and His161 and Ser248 may be important only for recognition of PZA.

In conclusion, the present study identifies PZA as an inhibitor of MtQAPRTase. Base on the structure of PZA new inhibitors of MtQAPRTase could be designed. Because MtQAPRTase is a potential candidate target of new anti-TB drugs [5], [10]–[12], we expect that our present findings will contribute to their development.

## Supporting Information

Figure S1 **The possible confirmations of His tag.** The ribbon model (A) and surface model (B) of WT MtQAPRTase (PDB ID:1QPO) and 3 calculated structures, after elongating six histidines in C-terminal and molecular dynamics simulation, were shown. The one subunit is depicted in pale cyan while the other subunit is depicted in green. The additional six histidines are depicted in red. The active site of MtQAPRTase is shown in a broken line.
(TIF)
