# Supplementary figures and images for: Biochemical Characterization of Quinolinic Acid Phosphoribosyltransferase from Mycobacterium tuberculosis H37Rv and Inhibition of Its Activity by Pyrazinamide
Source: PLoS One. 2014 Jun 20;9(6):e100062. doi: 10.1371/journal.pone.0100062 (PMC4065032; doi:10.1371/journal.pone.0100062)

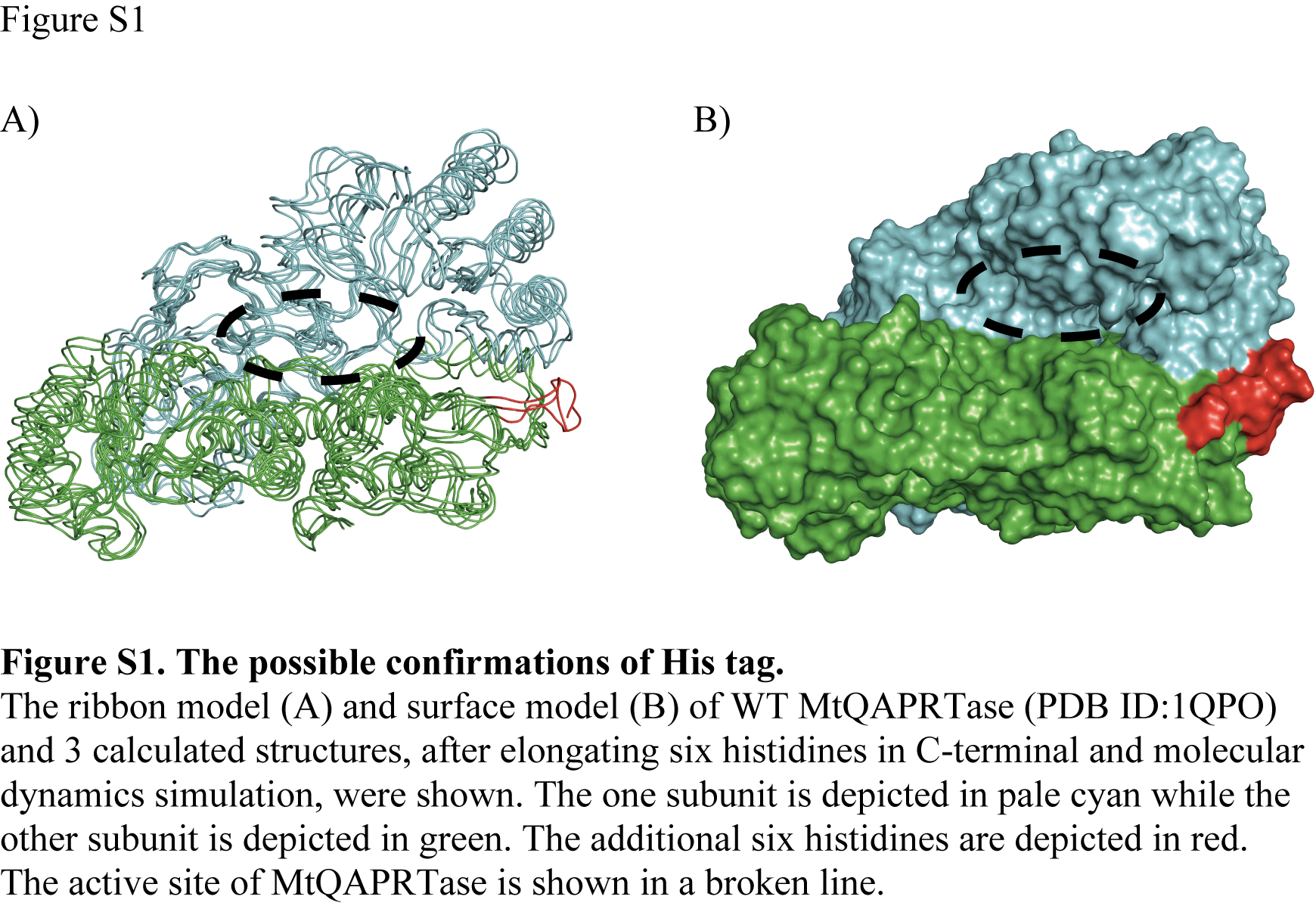

Supplement: Figure S1 — The possible confirmations of His tag. The ribbon model (A) and surface model (B) of WT MtQAPRTase (PDB ID:1QPO) and 3 calculated structures, after elongating six histidines in C-terminal and molecular dynamics simulation, were shown. The one subunit is depicted in pale cyan while the other subunit is depicted in green. The additional six histidines are depicted in red. The active site of MtQAPRTase is shown in a broken line. (TIF) [file pone.0100062.s001.tif]
